# Dosimetric and feasibility evaluation of a CBCT‐based daily adaptive radiotherapy protocol for locally advanced cervical cancer

**DOI:** 10.1002/acm2.13783

**Published:** 2022-10-08

**Authors:** Daniela Branco, Jyoti Mayadev, Kevin Moore, Xenia Ray

**Affiliations:** ^1^ Department of Radiation Medicine and Applied Sciences University of California San Diego 3855 Health Sciences Drive, #0865 La Jolla California USA; ^2^ California Protons Cancer Therapy Center San Diego California United States

**Keywords:** adaptive radiotherapy, cervical cancer, cone‐beam CT, dosimetry, image‐guided radiotherapy, target margins

## Abstract

**Purpose:**

Evaluate a cone‐beam computed tomography (CBCT)‐based daily adaptive platform in cervical cancer for multiple endpoints: (1) physics contouring accuracy of daily CTVs, (2) CTV coverage with adapted plans and reduced PTV margins versus non‐adapted plans with standard‐of‐care (SOC) margins, (3) dosimetric improvements to CTV and organs‐at‐risk (OARs), and (4) on‐couch time.

**Methods and materials:**

Using a Varian Ethos™ emulator and KV‐CBCT scans, we simulated the doses 15 retrospective cervical cancer patients would have received with/without online adaptation for five fractions. We compared contours and doses from SOC plans (5–15 mm CTV‐to‐PTV margins) to adapted plans (3 mm margins). Auto‐segmented CTVs and OARs were reviewed and edited by trained physicists. Physics‐edited targets were evaluated by an oncologist. Time spent reviewing and editing auto‐segmented structures was recorded. Metrics from the CTV (D99%), bowel (V45Gy, V40Gy), bladder (D50%), and rectum (D50%) were compared.

**Results:**

The physician approved the physics‐edited CTVs for 55/75 fractions; 16/75 required reductions, and 4/75 required CTV expansions. CTVs were encapsulated by unadapted, SOC PTVs for 56/75 (72%) fractions—representative of current clinical practice. CTVs were completely covered by adapted 3 mm PTVs for 71/75 (94.6%) fractions. CTV D99% values for adapted plans were comparable to non‐adapted SOC plans (average difference of −0.9%), while all OAR metrics improved with adaptation. Specifically, bowel V45Gy and V40Gy decreased on average by 87.6 and 109.4 cc, while bladder and rectum D50% decreased by 37.7% and 35.8%, respectively. The time required for contouring and calculating an adaptive plan for 65/75 fractions was less than 20 min (range: 1–29 min).

**Conclusions:**

Improved dose metrics with daily adaption could translate to reduced toxicity while maintaining tumor control. Training physicists to perform contouring edits could minimize the time physicians are required at adaptive sessions improving clinical efficiency. All emulated adaptive sessions were completed within 30 min however extra time will be required for patient setup, image acquisition, and treatment delivery.

## 1 INTRODUCTION

In the United States, 13 800 cases of invasive cervical cancer were diagnosed in 2020^1^ and these cases were most commonly diagnosed in women aged between 35–44.^2^ The standard‐of‐care (SOC) for the curative management of locally‐advanced cervical cancer consists of chemoradiation followed by brachytherapy, with expected cure rates of 30%–90% depending on the stage.^3^ Chemoradiation of the pelvic region, however, has been associated with severe (grade 3–4) gastrointestinal and genitourinary late toxicity rates occurring 6%–11%,^4^ often creating complications such as malabsorption, incontinence, and fistulae in young patients.

The recently increased usage of intensity‐modulated radiotherapy (IMRT) for cervical cancer has allowed for highly conformal radiation doses to the target volumes (cervix, uterus, parametrium, and pelvic lymph nodes) with improved sparing of organs‐at‐risk (OAR) and decreased toxicity.^5^, ^6^, ^7^, ^8^ However, treatment plans are typically designed based on a computed tomography (CT) scan acquired several weeks before treatment. In addition, the treatment is delivered over 5–6 weeks, while the pelvic anatomy is prone to considerable positional and volumetric changes over this timeframe.^9^, ^10^, ^11^, ^12^, ^13^ As a result, clinical target volumes (CTV) and OARs contoured during planning may differ substantially from the anatomy‐of‐the‐day during treatment. This can lead to healthy tissue receiving unintended doses of radiation and/or portions of the target being underdosed.

These anatomical changes range from 1–3 cm^14^, ^15^ and must be accounted for^16^, ^17^ in CTV‐to‐PTV margins. Traditional SOC margins of 15 mm for the uterus and cervix, 10 mm for the vagina and parametria, and 5–7 mm for the nodal CTV are widely used.^18^, ^19^, ^20^ Consequently, substantial normal tissue volume is also included in the PTV and exposed to prescription doses, thus increasing toxicity risks.^21^ To reduce geometric uncertainty, image‐guided radiotherapy (IGRT) was developed and widely adopted in clinical practice.^22^ While IGRT improves patient alignment prior to treatment, the current practice utilizes the same treatment fields every day irrespective of the day‐to‐day internal anatomic changes. Adaptive radiotherapy (ART) takes the concept of IGRT one step further, by using daily imaging to evaluate anatomical changes and modifying the treatment plans accordingly.

The new Ethos™ platform (Varian Medical Systems, Palo Alto, CA) allows for daily online adaptation of the target and OAR contours based on daily cone‐beam computed tomography (CBCT) imaging.^23^ It uses automated re‐planning to quickly create an optimized plan of the day for each treatment fraction. Initial reports demonstrated that Ethos' online ART workflow for prostate cancer improved CTV D98% and reduced normal tissue doses^24^ as well as showing the general feasibility of the Ethos adaptive platform for pelvic cases (bladder, rectum, anal, and prostate).^27^, ^30^ The ability to quickly adapt a treatment for the anatomy‐of‐the‐day could ensure target coverage at each fraction and potentially eliminate the need for the large margins used in cervical cancer radiotherapy, thus decreasing normal tissue toxicity. However, using a daily adaptive protocol also requires substantial extra clinical resources as treatment times are lengthened, and expert contour and plan review are needed. The purpose of this study is to evaluate a daily adaptive protocol using the Ethos platform in locally‐advanced cervical cancer for multiple endpoints: (1) contouring accuracy of daily CTVs, (2) CTV coverage with adapted reduced PTV margins versus non‐adapted SOC margins, (3) dosimetric improvements to the CTV and OARs with adapted reduced PTV margins versus non‐adapted SOC margins, and (4) on‐couch time.

## 2 METHODS

To conduct this study, we simulated the Ethos steps for adaptive treatment planning and delivery using an Ethos “emulator.” The emulator is an exact representation of the real system with the only limitation being the impossibility to deliver the plan created. It uses the same auto‐segmentation and auto‐planning algorithms as a clinical Ethos v1.0 but can be applied to retrospectively capture CBCTs, allowing offline evaluation of workflows and system performance without real treatment deliveries. The on‐couch adaptive workflow implemented with Ethos starts with the acquisition of a kV‐CBCT that is used for AI auto‐segmentation of structures referred to as “influencer” structures. Influencer structures are organs near the target that impact its shape and position. For cervix cases, Ethos influencers are the bladder, bowel, rectum, and uterus. After the user edits and approves the influencer contours, they are used to guide the auto‐segmentation of the targets. After the targets are reviewed and approved, the adapted plan is optimized using the same planning goals that were used to create the initial plan. A synthetic CT (sCT), generated by deforming the simulation CT based on that day's CBCT, is used as the image for dose calculation.^28^ Adapted targets and OARs are superimposed on the sCT to obtain DVH metrics. The original treatment fields for the patient are also re‐calculated on the sCT to compare the non‐adapted to the adapted plan's dose.

**FIGURE 1:**
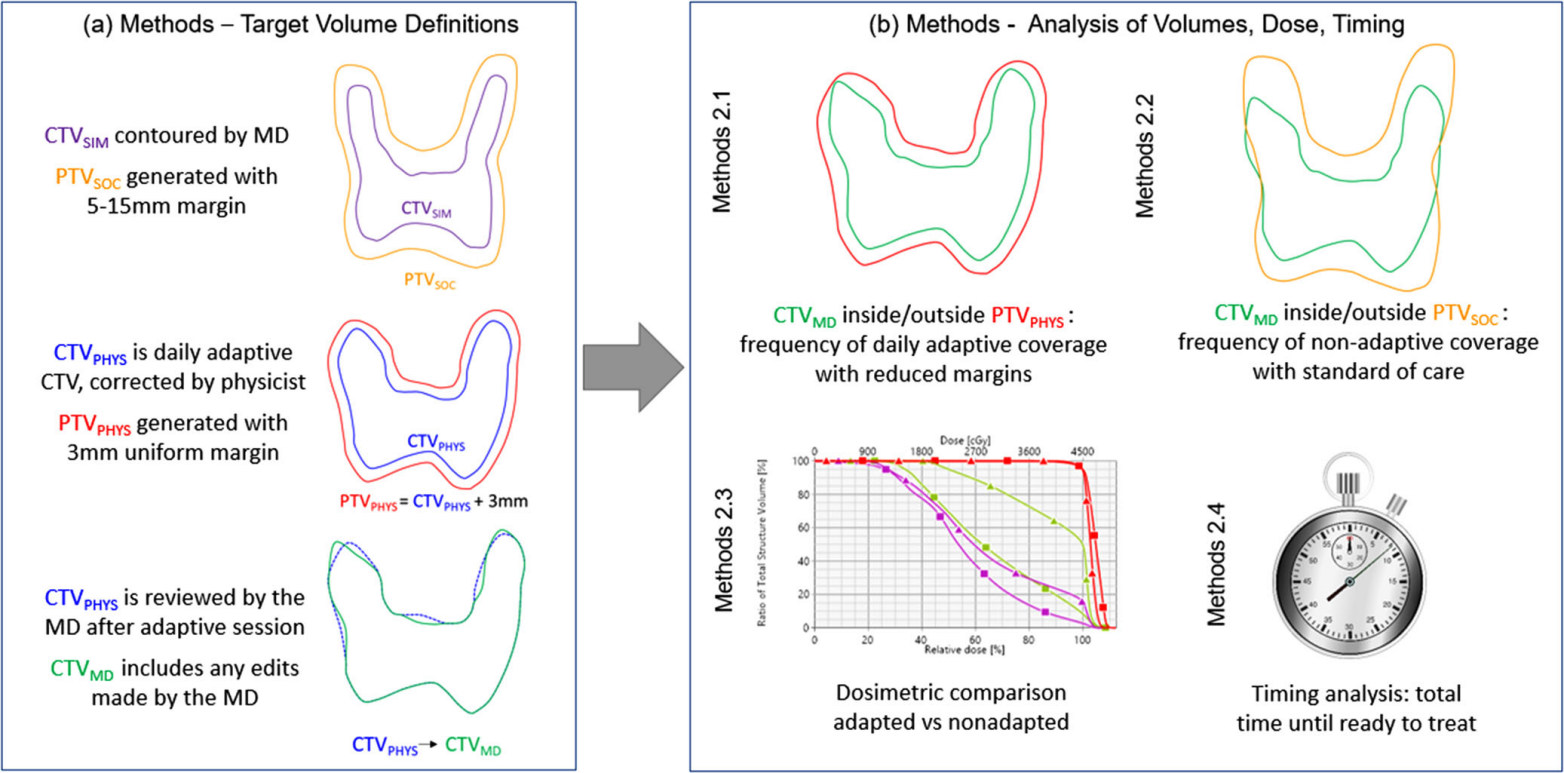
Diagram representation of the CTVs and PTVs generated and evaluated

The simulation CT and five on‐treatment CBCTs of 15 retrospective cervical cancer patients were used in this study (IRB# 200135). Figure 1a shows a diagram describing the multiple CTVs and PTVs used in our analysis. The 15 patients’ CT scans were imported into the emulator and re‐planned using the same structure set from the clinical plans except with a 3 mm symmetric CTV‐to‐PTV margin. Each physicist re‐planned five patients to create the prioritized list of dose goals that Ethos uses to auto‐plan subsequent adapted plans. All plans used 12‐field IMRT and dosimetrically met our institutional guidelines for cervical cancer irradiation. 12‐field IMRT was used because it substantially speeds up the optimization time for adapted plans compared to VMAT, from YY minutes to XX minutes. Faster dose optimization and calculation are important for adaptive workflows because the patient is on the couch the entire time that the plan is being created and their internal anatomy may be changing (e.g., bladder filling). Thus, reducing the time required to adapt as much as possible is key to both patient comfort and acceptability of the adapted plan.

Five fractions for each patient were then adapted in the emulator using their clinically‐acquired kV‐CBCT scans to simulate Ethos’ daily auto‐segmentation and auto‐planning workflow. The five CBCTs were from five different treatment days evenly distributed through their range of treatments, in order to represent expected changes in anatomy (e.g., bowel/rectum/bladder changes and/or tumor shrinkage). During each simulated fraction, auto‐segmented structures were reviewed and edited by the physicist and, if necessary, contouring edits to OARs and CTVs (CTV_PHYS_) were made. Then, 3 mm margins were added to CTV_phys_ to generate PTV_phys._ To ensure adequate training in cervical cancer CTV delineation, each participating physicist watched a training video produced by our institution prior to simulating the adaptive sessions. Once structure edits were completed, adapted plans were generated and doses were calculated for the anatomy‐of‐the‐day. These plans were mock‐delivered by the emulator to simulate full adaptive sessions and thus allowing us to capture dose and timing data.

All physicist‐adapted targets were reviewed by an experienced gynecologic radiation oncologist for accuracy and, when necessary, CTV_PHYS_ were corrected to create CTV_MD_. This sequence of steps mimics a potentially efficient clinical workflow that minimizes the time a physician is needed at daily adaptive fractions.

Current non‐adapted SOC plans use CTV‐to‐PTV margins of 15 mm for the uterus and cervix, 10 mm for the vagina and parametria, and 5 mm for the nodal CTV (PTV_SOC_). To compare what the patients would receive with current SOC non‐adapted delivery, we recalculated each patient's original clinical plan (all with 3‐arc VMAT delivery) onto the five sCTs and the anatomy‐of‐the‐day.

After generating the adapted and un‐adapted volumes and doses, four analyses were performed as described below (Figure 1b):

### 2.1 Physicist contouring accuracy of daily CTVs

To evaluate the quality of the physicists’ contour review and edits we assessed (1) how often the physician corrected CTV_PHYS_ to create CTV_MD_, and (2) whether CTV_MD_ was fully encapsulated by PTV_PHYS_ (Figure 1b). Physician contouring corrections were classified as reductions (shrinking CTV_PHYS_) or expansions (increasing CTV_PHYS_). The volume of these corrections was also measured.

### 2.2 CTV coverage frequency

To compare with our current clinical practice, we determined the number of fractions where CTV_MD_ was included within PTV_SOC_ (Figure 1b) without adaptation (real clinical scenario). This was performed by registering each kV‐CBCT to the planning CT and copying the original PTV_SOC_ to the daily image for comparison to the anatomy‐of‐the‐day. Automatic image registration focused on bony anatomy was performed and then manually adjusted as needed. The volume of CTV_MD_ extending outside PTV_SOC_ was also measured.

### 2.3 Dosimetric comparison of CTV and OARs

Twelve out of 15 patients had the same prescribed dose (180cGy × 25 fractions) and were used in the dosimetric analysis. The impact of adaptation on target dose was evaluated by comparing CTV_MD_ D99% (%) for non‐adapted SOC plans versus adapted plans. OAR sparing was compared for the true bowel (V45Gy(cc), V40Gy(cc)), bladder (D50%(cGy)), and rectum (D50%(cGy)).

Auto‐segmented bowel (Ethos bowel) required considerable contouring edits which were performed offline, after the simulated treatment, to create the true bowel. These edits were considered irrelevant for adaptive plan optimization because they were far from the target but were performed afterward to obtain accurate dose metrics for comparison. This workflow helped minimize the time required at each adaptive session, while still obtaining high‐quality treatment plans. Volume differences between the Ethos and true bowels were recorded.

### 2.4 On‐couch time

For each adaptive session, the physicist recorded the time spent reviewing and editing OARs, reviewing and editing CTVs, and the total time until ready to treat. The total included the time required for auto‐segmentation, contour review/edits, re‐optimization, and dose calculation. It did not include the time that would be required for patient setup, image acquisition, plan QA, or beam delivery.

## 3 RESULTS

### 3.1 Physicist contouring accuracy of daily CTVs

**FIGURE 2:**
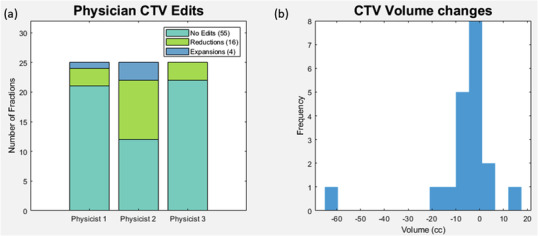
(a) Frequency and (b) volume of corrections made by the physician to create CTV_MD_ from the CTV_PHYS_ (edited by physicist). The majority of CTV_PHYS_ required no further edits by the physician. When edits were needed, they generally were made to reduce the volume. Overall median volume edits were small but ranged from 0.6–63.3 cc

**FIGURE 3:**
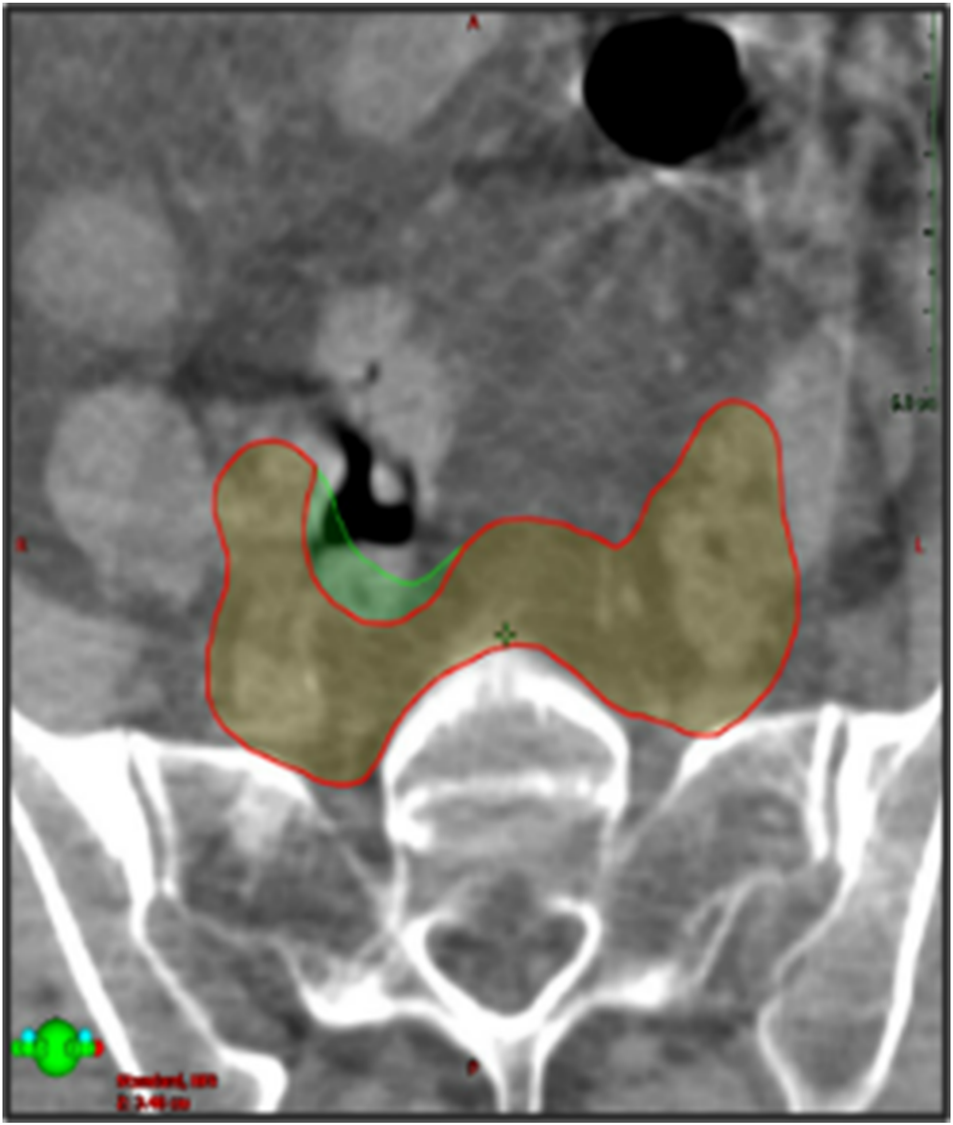
Example of a CTV edit by a physician, where they edited CTV_PHYS_ (green) to CTV_MD_ (red) to avoid a bowel loop

Figure 2a shows how often CTV_PHYS_ required further corrections by the physician to generate CTV_MD,_ and Figure 2b shows the changes in volume when edits were made. For most simulated treatments (55/75 fractions) the physician had no edits to physicist‐edited CTV_PHYS_; for 16/75 fractions the physician reduced CTV_PHYS_ to create CTV_MD_; only 4/75 fractions required expanding CTV_PHYS_. The median change in CTV_PHYS_ volume when the physician expanded it was 5.5 cc [0.6–16.9 cc]. The median change in CTV_PHYS_ volume when the physician reduced it was 9.5 cc [3.2–63.3 cc]. Figure 3 shows an example of a physician CTV_PHYS_→CTV_MD_ reduction edit.

While the physician made edits to CTV_PHYS_ on 25/75 fractions, CTV_MD_ was completely covered by the adapted PTV_PHYS_ in 71/75 (94.6%) fractions. However, the four fractions where CTV_PHYS_ was expanded to create CTV_MD_ were not fully encapsulated by PTV_PHYS_, and thus were larger than 3 mm corrections. The volume of CTV_MD_ extending outside PTV_PHYS_ was measured and found to have a median value of 2.9 cc [0.1–9.2 cc].

### 3.2 CTV coverage frequency with SOC

**FIGURE 4:**
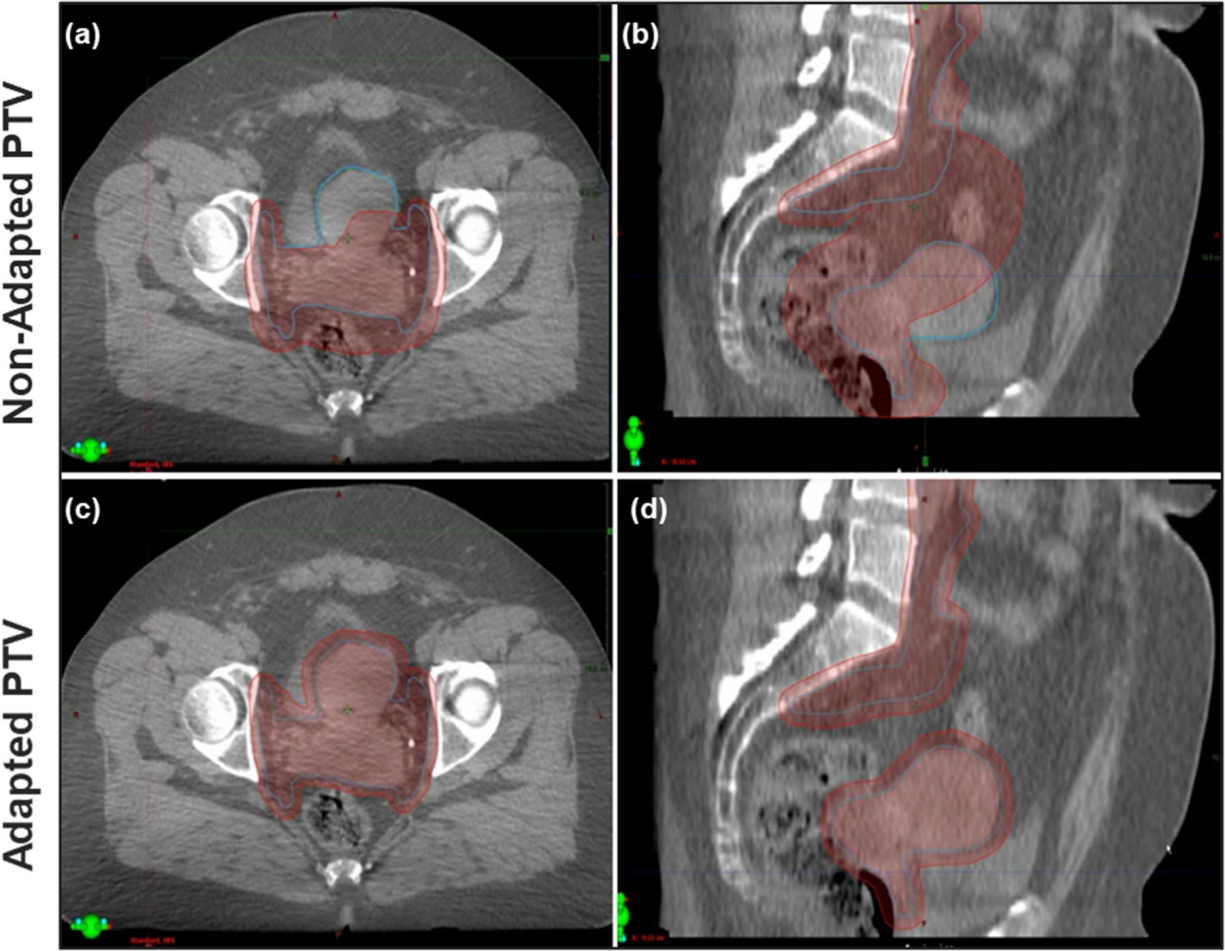
Example showing the anatomy‐of‐the‐day CTV_MD_ (blue) outside of the non‐adapted PTV_SOC_ (red), shown in (a) and (b). This means the CTV on the day of treatment would be under‐dosed if no adaptation was performed. Also, bowel and rectum were included in the non‐adapted PTV_SOC_ receiving prescription dose in (a) and (b). The adapted PTV_PHYS_ in red, in (c) and (d), encompasses the CTV_MD_ on the day of treatment and substantially spares the rectum and bowel

CTV_MD_ was completely encapsulated by PTV_SOC_ for only 19/75 (25.3%) of fractions. The volume of CTV_MD_ extending beyond PTV_SOC_ had a median value of 1.3 cc [0.1–24.1 cc]. Figure 4 shows an instance of substantial CTV_MD_ extending outside of PTV_SOC_. In addition to underdosing the anterior CTV_MD_, a large portion of the rectum and bowel were also included in that day's irradiation volume.

### 3.3 Dosimetric improvements to CTV and OARs

**FIGURE 5:**
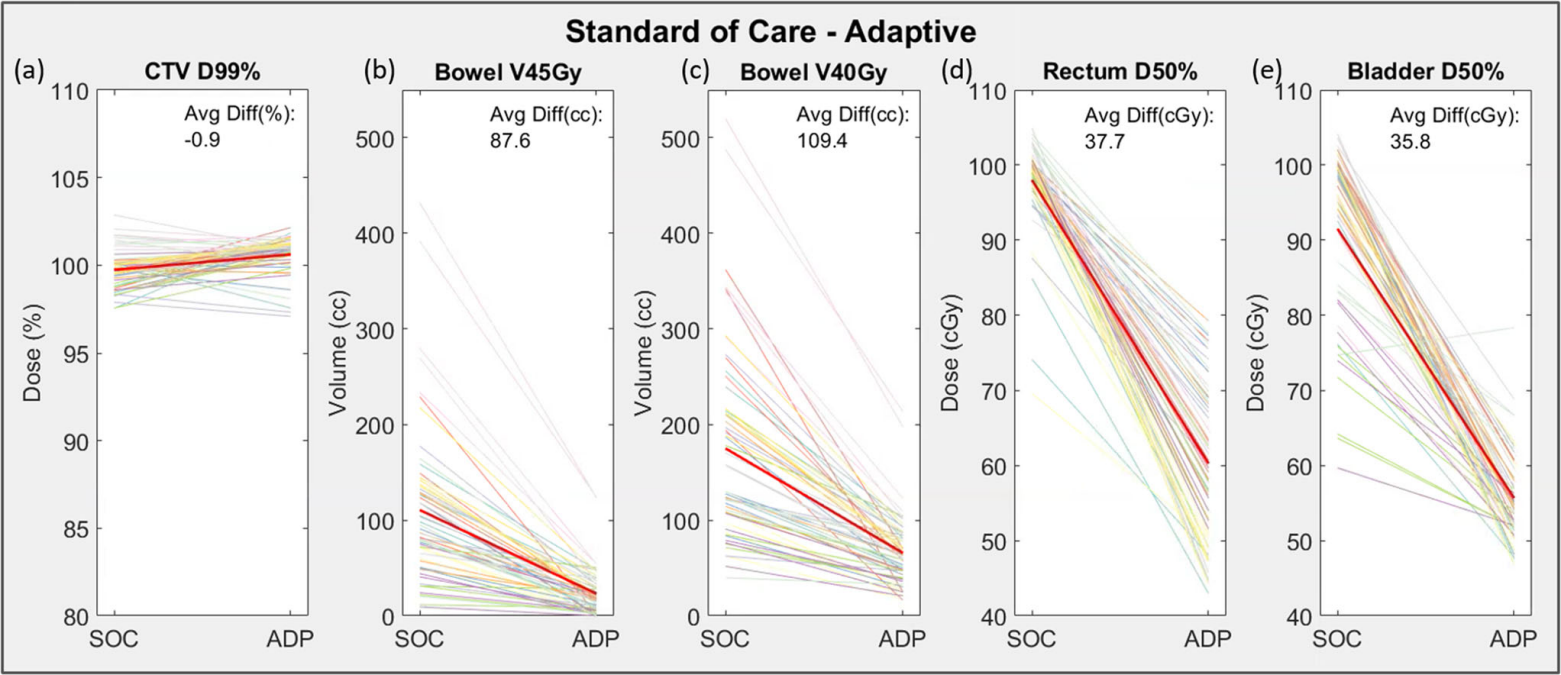
Dosimetric improvement of adapted plans in comparison to SOC plans. Each patient is represented by a separate color with the overall average represented by the bold red line. An ascending/descending line represents an improvement in the CTV/OAR metric respectively for a single fraction. CTV coverage was comparable as measured by D99%, while all OAR metrics improved with adaptation. SOC, standard‐of‐care

Figure 5 compares dose metrics between adapted plans using 3 mm PTV margins and non‐adapted SOC plans. Target coverage (CTV_MD_ D99%) for adapted plans was comparable to non‐adapted SOC plans (average difference of −0.9%). OAR metrics improved with adaptation at every fraction, save one where bladder D50% increased. On average, bowel V45Gy and V40Gy, measured from the corrected bowel contours, improved by an average of 87.6 and 109.4 cc, respectively. Bladder and rectum D50% reduced on average by 37.7% and 35.8%, respectively. A boxplot of each patient's individual values for both plans is available in Supporting Information.

The auto‐segmented Ethos bowel was consistently smaller in volume than the true bowel (57/60 fractions) by an average of 73.8 cc ± 102.2 cc. This impacted DVH metrics from unadapted plans by increasing V45Gy on average by 28.6 cc (82 cc for Ethos bowel versus 110.6 cc for true bowel) and increasing V40Gy on average by 38.5 cc (136.6 cc for Ethos bowel versus 175.1 cc for true bowel). For adapted plans, a similar trend was seen where V45Gy increased on average by 13.0 cc (10.0 cc for Ethos bowel versus 23.0 cc for true bowel) and V40Gy increased by 22.3 cc (43.4 cc for Ethos bowel vs. 65.7 cc for true bowel).

### 3.4 On‐couch time

Table 1 shows the times recorded for reviewing and editing OARs and CTVs, along with the total time until ready to treat. Most fractions (65/75) were completed in less than 20 min and only 2/75 required more than 23 min (treatment time not included).

**TABLE 1:** Time spent editing OARs and CTVs after Ethos performs its auto segmentation and the total time from acquisition of CBCT until ready to start the treatment

	**Average time reviewing/editing OARs (min)**	**Average time reviewing/editing CTVs (min)**	**Average time until ready to treat (min)**
Physicist 1	4 [1–13]	7.9 [2.5–12.5]	16.8 [9.5–29]
Physicist 2	3.7 [1–10]	6.3 [1.5–13]	17.1 [7–22]
Physicist 3	3.3 [1–12]	8 [3–12]	16.6 [7.5–32]
**Total time**:	**3.6 [1**–**13]**	**7.4 [1.5**–**13]**	**16.8 [7**–**29]**

## 4 DISCUSSION

Current radiotherapy SOC target margins for cervical cancer patients include large 5–15 mm CTV‐to‐PTV margins to account for the substantial organ motion observed in the area. However, we found that even with these large margins, the CTV_MD_ at treatment was completely encapsulated by PTV_SOC_ for only 25.3% of fractions, although the amount of CTV_MD_ extending outside PTV_SOC_ was generally very small (average = 1.3 cc). This result corroborates numerous other studies^14^ that found large 2–3 cm margins would be required to completely account for inter‐fraction target motion in some patients. Additionally, this result suggests that in addition to a normal tissue benefit from adaptation with smaller margins, we could also see an increase in tumor control by ensuring that at every fraction the CTV V100% ≥ 100%. Additionally, analyses of Ethos plan quality have demonstrated improved dose metrics for the auto‐generated IMRT plans versus VMAT plans for pelvic targets.^25^, ^26^

In the adaptive workflow proposed here, CTV_MD_ was encapsulated by PTV_PHYS_ for 94.6% of fractions, while bowel, rectum, and bladder dose metrics were simultaneously improved. As outlined in QUANTEC, there is a strong correlation between bowel toxicity and the absolute amount of bowel receiving ionizing doses of radiation, with bowel complication probability models using bowel V40Gy and V45Gy as highly predictive dosimetric surrogates.^28^, ^29^ We found in our study that bowel V45Gy and V40Gy were reduced on average from 110.6 to 23 cc and 175.2 to 65.7 cc, respectively, potentially leading to a reduction in GI toxicities. The largest gains were expected to be seen in bowel metrics, however, adaptation also substantially improved D50% doses for the rectum and bladder from 98% to 60.2% and 91.5% to 55.6%, respectively. These normal tissue dose reductions could potentially lead to decreases in urinary and late rectal toxicities if online ART was used prospectively. It is presumed that these gains were largely due to the substantial decrease in margins with ART, but are also attributable to the improved conformality of adapted plans to the edited CTV/PTV.

Aside from observing coverage and dosimetric improvements in both target and OARs, this work also showed that physicists can be trained to evaluate and correct auto‐segmented gynecological CTVs using the physician‐drawn CTVs from the initial plan as their guide. Other clinics have trained different non‐physicians to fulfill this adapter role, such as dosimetrists or therapists. Our workflow incorporated a physicist as the adapter due to having more experience and background in both contouring and planning compared to a therapist, therefore being more equipped to make fast decisions on target contours while patients are on the table. Additionally, because a new plan is optimized for every treatment, a physicist will always be present at each treatment to review the plan, and thus is also available to help create it. In our study, this approach led to no further CTV edits required by the physician for a majority (73.3%) of fractions and most physician edits were made to reduce the target volume. Edits that expanded the CTV volume were required only 5.4% of the time. Thus, there is a very low risk of target miss even when a trained physicist adjusts the contours at each fraction instead of a physician. Having a physicist available at the machine for the bulk of contour edits reduces the time a physician's presence is required at each fraction, relieving this potential burden on the physician's clinical schedule. Instead, they could be paged for the final minutes of contour edits to review and make further edits as needed, and after several successful fractions could review offline and provide additional guidance for the next treatment when needed.

The feasibility of the proposed workflow also extends to the amount of time needed per fraction. Adaptive treatment sessions in this study were 16.8 min on average, although this does not include other necessary time for patient set‐up, CBCT image acquisition, plan QA, and beam delivery. Based on our clinical experience we anticipate these additional steps may require 15–20 min and thus most adapted sessions could fit into a total treatment slot of 30–40 min. This is on par with the time we dedicate to most SBRT treatments at our clinic and is thus feasible. However, one challenge we could not address in this study is the intra‐fraction anatomical changes (e.g., bladder filling) likely to occur during the segmentation and planning process. These changes can be evaluated by acquiring a second confirmation CBCT prior to treatment and implementing additional IGRT shifts if necessary.

**FIGURE 6:**
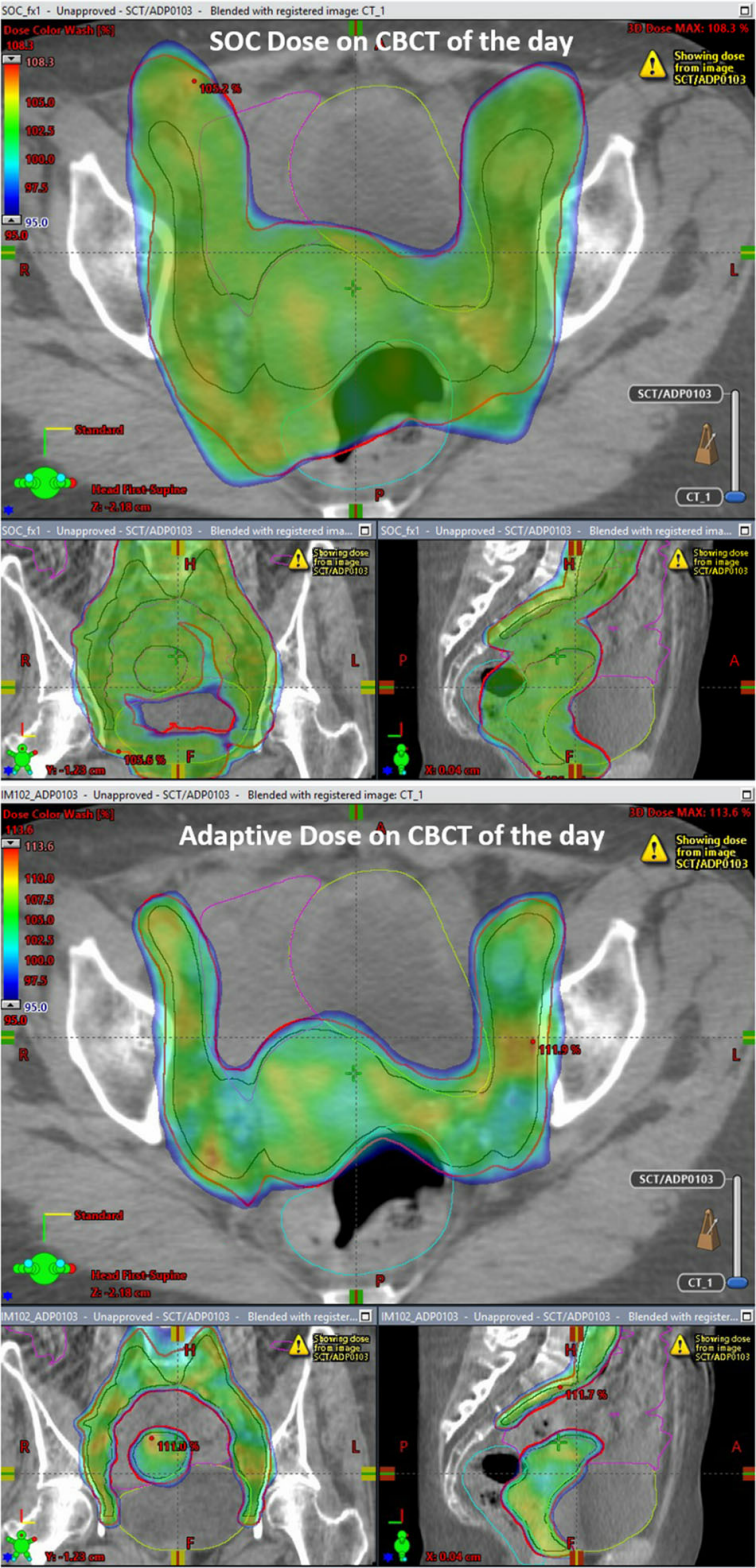
SOC planned dose (top) on the anatomy of the day (CBCT ‐ fraction 1) along with the original CTV_SIM_ and PTV_SOC_ created from the CT simulation scan. Adapted dose (bottom) also on the CBCT fraction 1, along with the CTV of the day determined by the physician, CTV_MD_, and PTV_PHYS_. Both CTVs were well covered by the 100% isodose line (CTV_SOC_ D99% = 101.3% and CTV_ADP_ D99% = 101.7%), but the larger margins of the SOC plan resulted in prescription dose extending into the bowel, rectum, and bladder, leading to inferior OAR metrics compared to the adapted plan. CBCT, cone‐beam computed tomography; SOC, standard‐of‐care

Some patients benefited from adaptation more than others. This can be observed in Figure 4 based on the slope of the plotted lines. Steep descending lines represent large improvements in OAR dose while shallower lines represent smaller improvements from adaptation. For instance, the patient represented with light purple (blue arrow in Figure 5, patient 8 in Figure S1), showed the largest bowel improvement. The mean bowel V45Gy volume decreased from 321.3 to 78.4 cc, (V40Gy from 396.3 to 149.2 cc, rectum D50% from 99.1% to 67.2% and bladder D50% from 88.1% to 52.5%) with ART. This result is largely attributable to the decrease in margins available with ART as shown in Figure 6. Figure 6 (top) shows the SOC planned dose overlaid onto the CBCT from fraction 1 along with the original CTV_SIM_ and PTV_SOC_. Figure 6 (bottom) shows the adapted dose overlaid onto the CTV of the day: CTV_MD_, and PTV_PHYS_. Both CTVs were well covered (CTV_SIM_ D99% = 101.3% and CTV_MD_ D99% = 101.7%), but the larger margins of the SOC plan delivered prescription dose extending into the bowel, rectum, and bladder.

**FIGURE 7:**
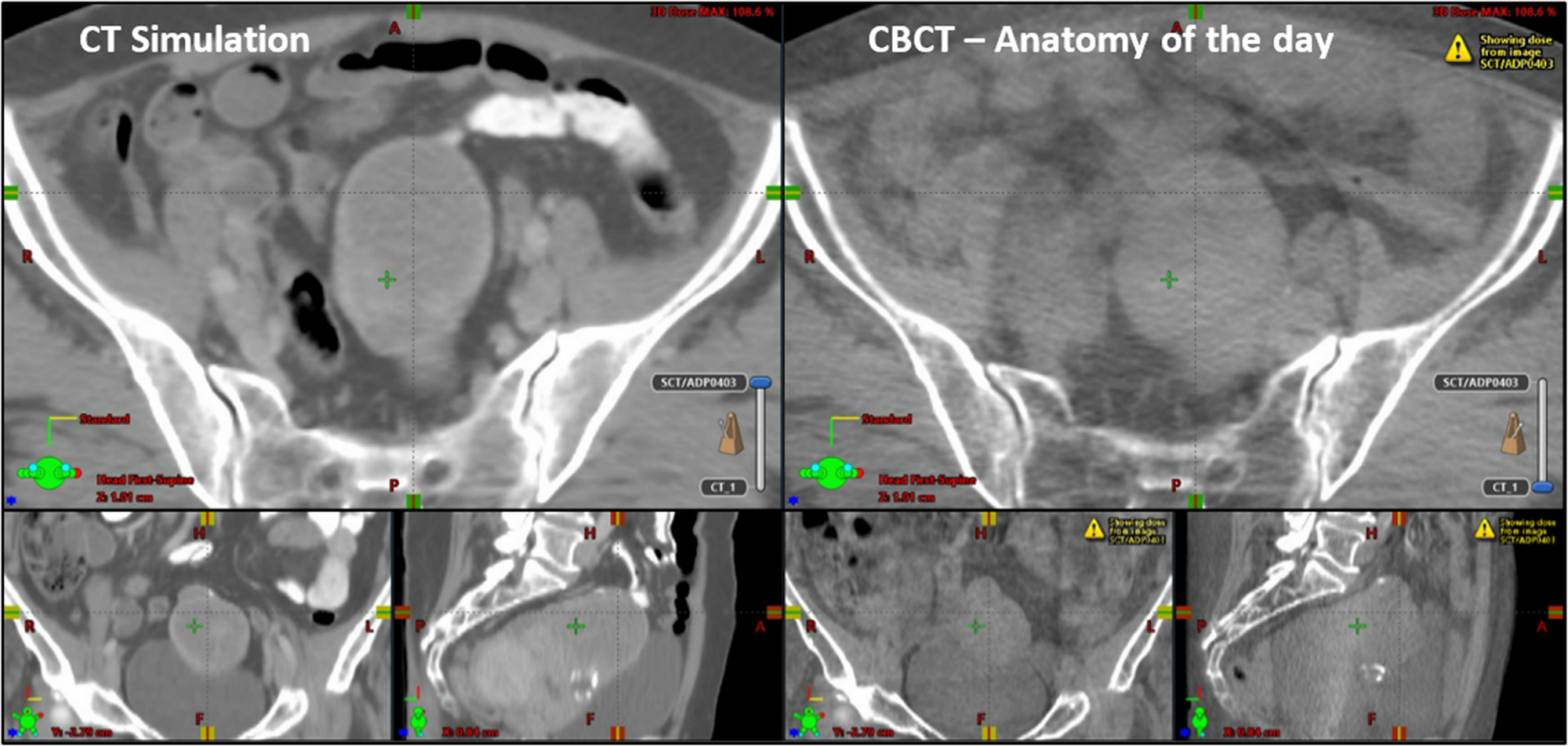
CT scan of patient 8 (left) and CBCT (right) 48 days later showing how some patients do not benefit extensively from adaptation due to their anatomy maintaining both shape and location through treatment. CBCT, cone‐beam computed tomography

In contrast, patient 11 showed the shallowest plotted lines in Figure 5 and therefore lowest improvements in dosimetric metrics. Figure 7 displays one of her CBCTs representative of the five CBCTs used in this study. Her target and OAR anatomy stayed consistent from CT simulation to that fraction (48 days in between) and thus she had little dosimetric benefit with adaption. Because of the extra clinical resources required for each adaptive fraction (time, personnel), it would be ideal to identify patients with large benefits prior to treatment and triage them for daily adaptive treatments while patients with less mobile anatomy continue with the current non‐adaptive SOC. Future studies will include examining the characteristics of those patients who show the largest benefit from adaptation and establishing whether or not we can predict patients with highly mobile anatomy prior to beginning treatment.

We also observed that some patients’ anatomies were not well differentiated in CBCT scans, either due to tissue composition or the presence of large amounts of gas leading to pronounced artifacts. Such patients may not be suitable candidates for adaptation. Additionally, elderly patients or those who cannot tolerate the treatment position for longer timeslots may also not be suitable for ART.

One limitation of this work was related to the Ethos bowel auto‐segmentation. The auto‐segmented bowels consistently had smaller volumes when compared to the manually corrected contours (true bowel). Corrections during each adaptive fraction were made to the Ethos bowel only when edits were needed near the CTV. The true bowel was created offline, and thus the true organ was not used during dose re‐optimization. This was considered a reasonable compromise between on‐couch treatment time and plan quality since most required edits were distant from the target being treated. This reasoning was validated with our data showing that, even though the true bowel was recorded to receive a higher V45Gy than the Ethos bowel values reported at the time of adaptation, all values were well within QUANTEC recommendations. Thus, spending the time to perfectly contour the bowel would have little impact on plan quality and patient safety. In addition, the true bowel with adaptation had substantially decreased V45Gy values compared to non‐adapted true bowel values thus demonstrating the dosimetric benefit of adaptation despite the volume underestimation. It is important to note that the observed improvements are a combination of the reduced margins and the use of adaptation. Reducing margins can only safely be done in the context of adaptation because of the increased certainty in target and OAR positioning at each fraction. Similarly, adapting without reduced margins in the context of cervical cancer is illogical due to the increased positioning certainty and known dose to the targets. The SOC margins are designed to help account for large inter‐fraction motion which is no longer a concern when the targets are being re‐drawn every day and the plan re‐optimized to those new targets. Thus, this work sought to evaluate the benefit from a clinical protocol using the margins we were planning to implement and report realistic dose changes.

## 5 CONCLUSION

We evaluated a daily online adaptive protocol on a retrospective cohort of 15 cervical cancer patients using the Varian Ethos CBCT‐based ART platform. We found that the CTV on the day of treatment was more frequently included in an adapted PTV with reduced margins, than the unadapted PTV with SOC margins. OAR dose metrics (bowel V45Gy, bowel V40Gy, rectum D50%, and bladder D50%) improved with adaptation. Thus, daily adaptation with reduced margins for cervical cancer could reduce toxicity, while maintaining the CTV dose (D99%). Trained physicists successfully edited CTVs during the simulated treatments and when reviewed by a physician, no further CTV edits were required for 55/75 fractions. The proposed workflow allows for team expertise integration into an efficient adaptive workflow. Further, most adaptive sessions for these patients would add only 20–30 min to the total treatment timeslot. Future studies will evaluate which patients are best suited for adaptation.

## CONFLICT OF INTEREST

Kevin Moore acknowledges consulting fees and honoraria from Varian Medical Systems.

## Supporting information

Supporting Information

Supporting Information

Supporting Information
