# Supplementary figures and images for: Dosimetric and feasibility evaluation of a CBCT‐based daily adaptive radiotherapy protocol for locally advanced cervical cancer
Source: J Appl Clin Med Phys. 2022 Oct 8;24(1):e13783. doi: 10.1002/acm2.13783 (PMC9859994; doi:10.1002/acm2.13783)

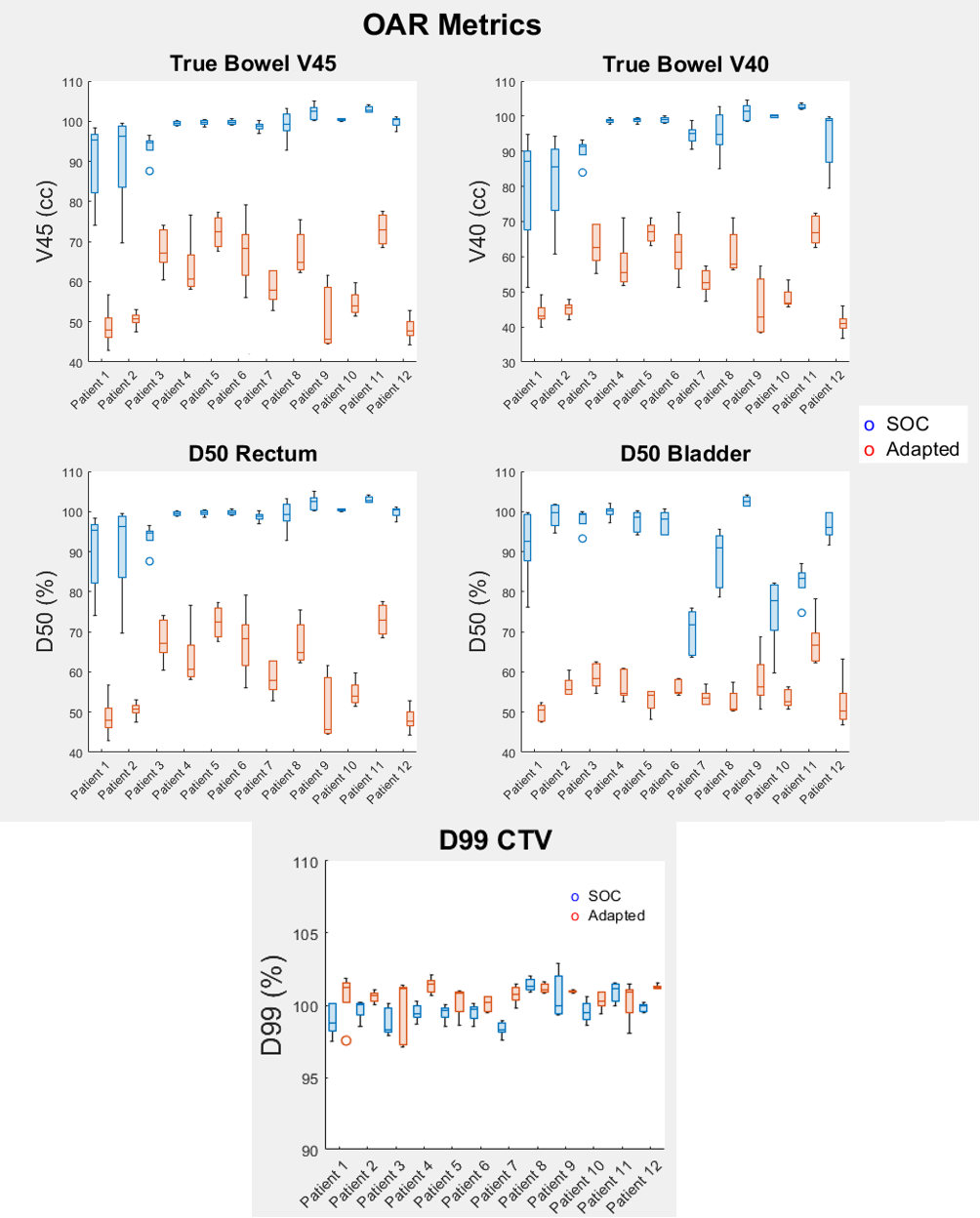

Supplement: Supplementary file 1 — Supporting Information [file ACM2-24-e13783-s002.png]

# OAR Metrics

## True Bowel V45

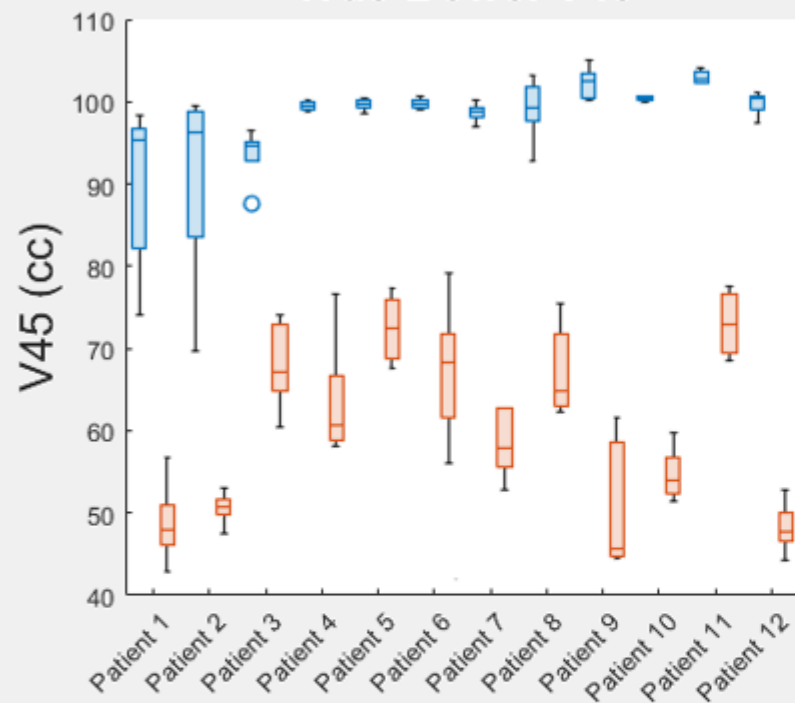

## True Bowel V40

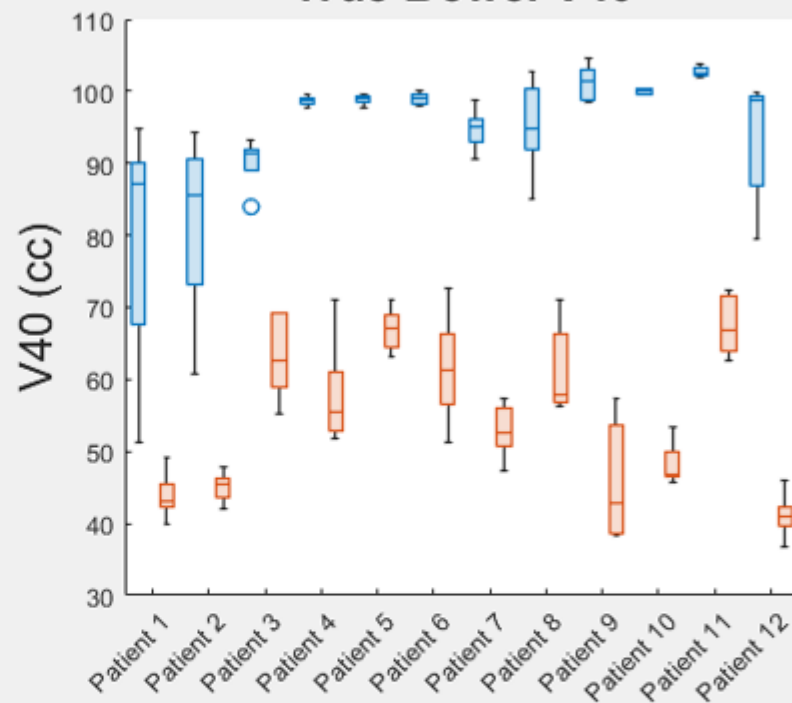

## D50 Rectum

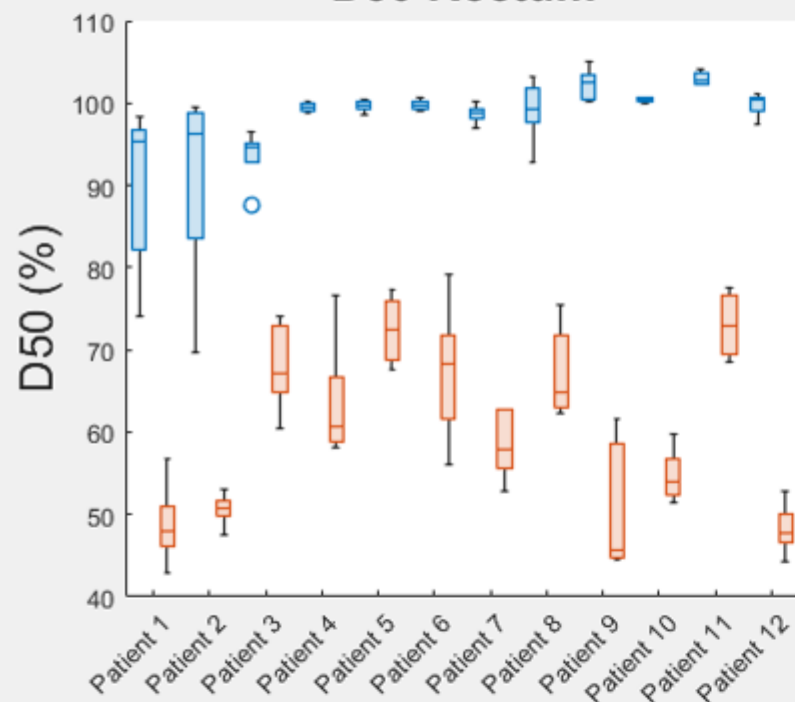

## D50 Bladder

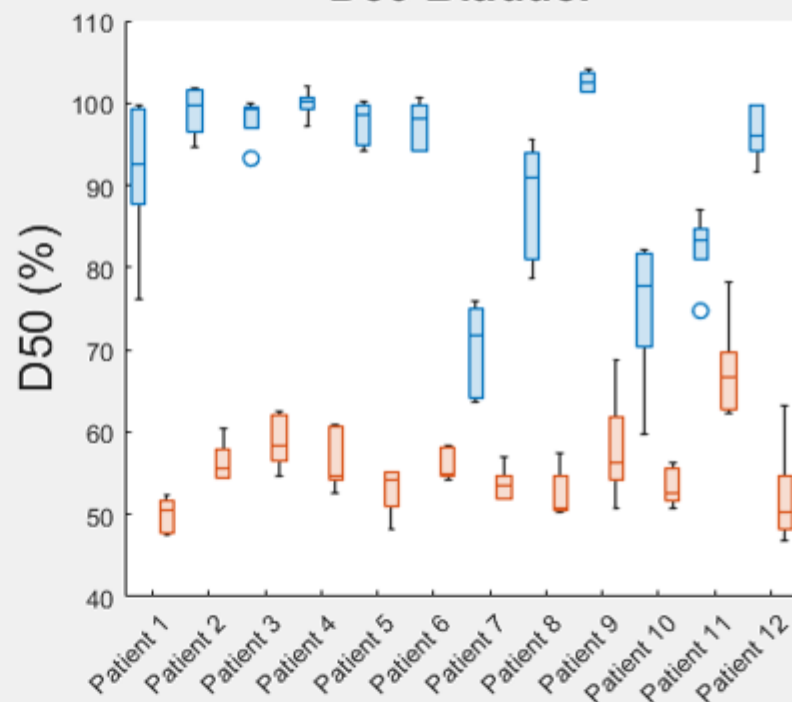

## D99 CTV

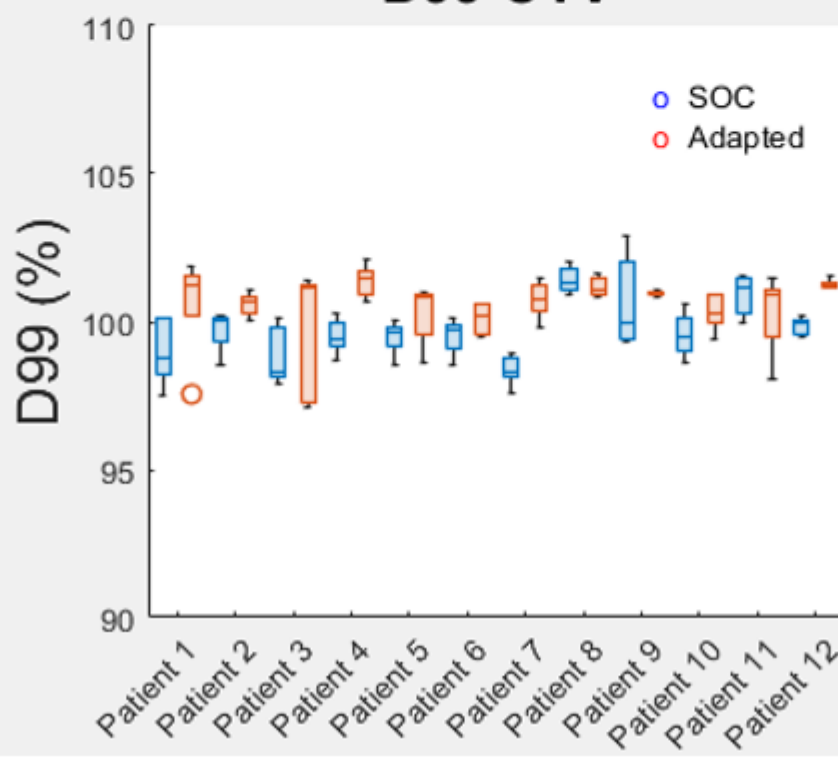

Supplement: Supplementary file 2 — Supporting Information [file ACM2-24-e13783-s001.pdf]
