# Supplementary material for: Dosimetric and feasibility evaluation of a CBCT‐based daily adaptive radiotherapy protocol for locally advanced cervical cancer
Source: J Appl Clin Med Phys. 2022 Oct 8;24(1):e13783. doi: 10.1002/acm2.13783 (PMC9859994; doi:10.1002/acm2.13783)
Supplement: Supplementary file 3 — Supporting Information [file ACM2-24-e13783-s003.docx]

Figure S1: Each patient’s dosimetry metrics for the SOC and adapted plans including all 5 CBCT fractions. Adaptation constantly showed a reduction in the OAR metrics, and therefore improved healthy tissue sparing. CTV coverage was kept nearly consistent through treatments, showing an improvement in the average value between all CBCT fractions on 10 out of 12 patients.
